# Supplementary material for: Stage-Specific Transcriptomic Insights into Seed Germination and Early Development in Camellia oleifera Abel
Source: Plants (Basel). 2025 Jul 24;14(15):2283. doi: 10.3390/plants14152283 (PMC12348211; doi:10.3390/plants14152283)
Supplement: Supplementary file 1 [file plants-14-02283-s001.zip › plants-3756124-supplementary.pdf]

**Supplementary materials**

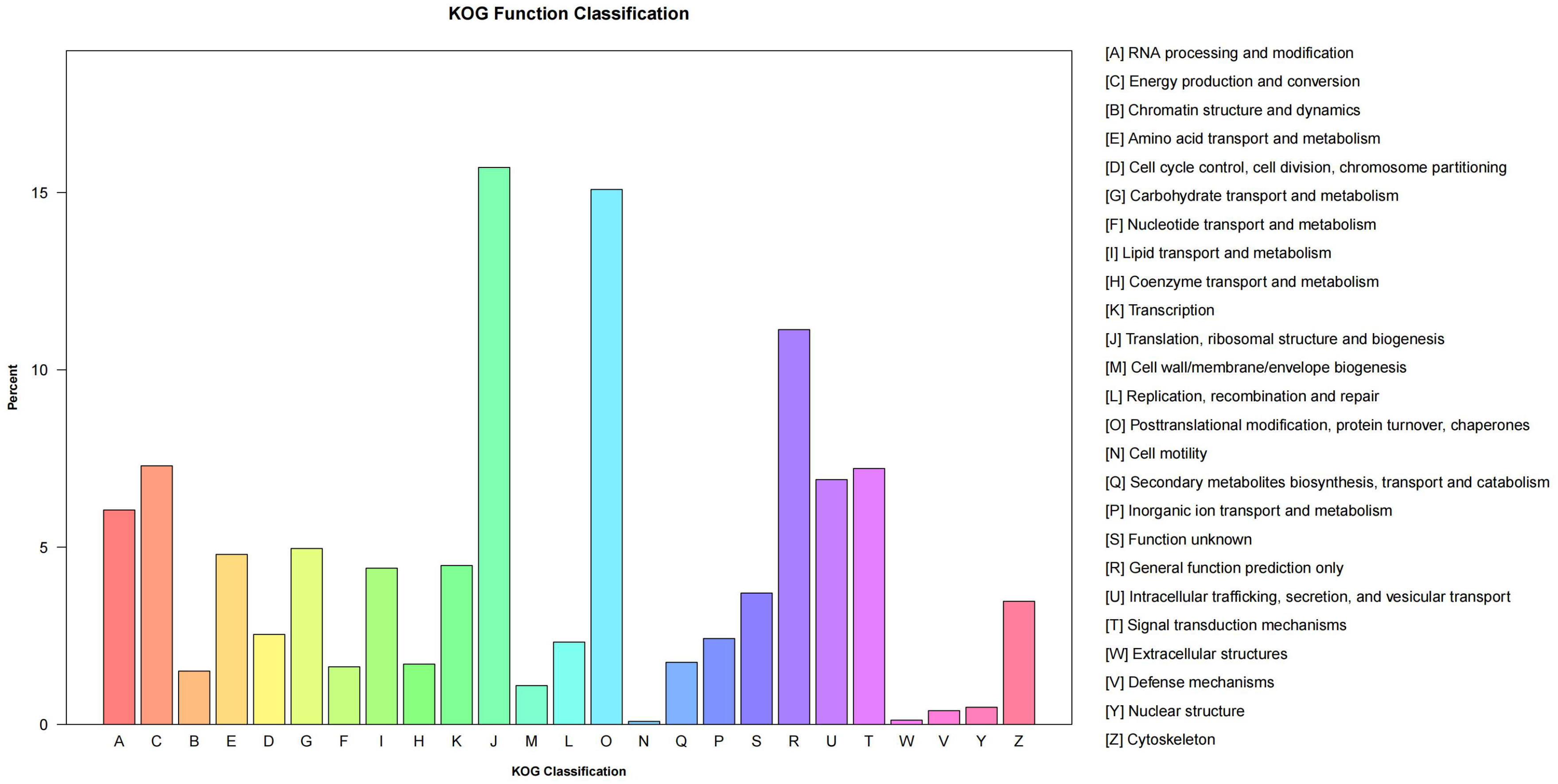


**Figure S1.** KOG functional classification of *C. oleifera* unigenes

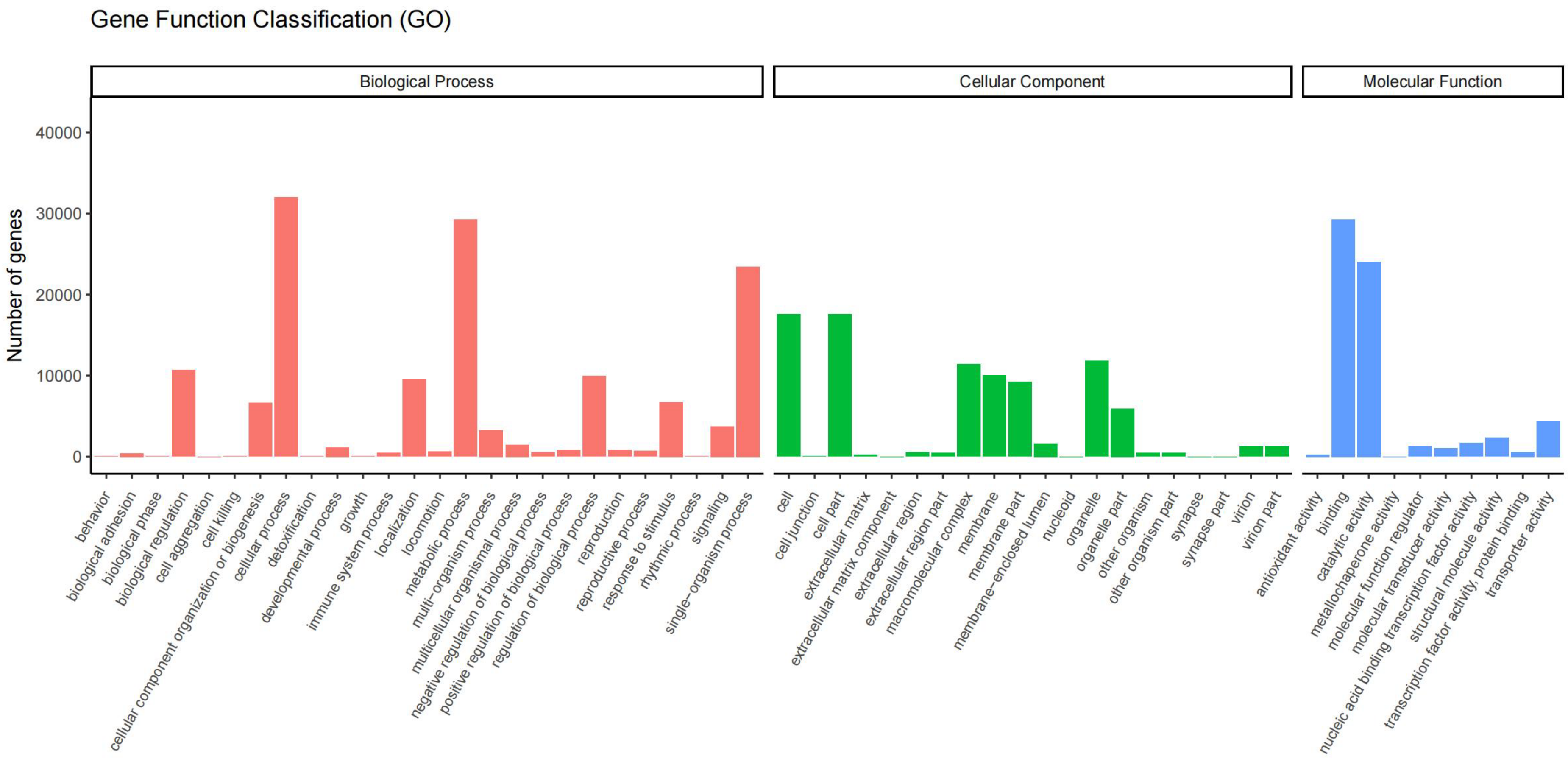


**Figure S2.** GO functional classification of *C. oleifera* unigene

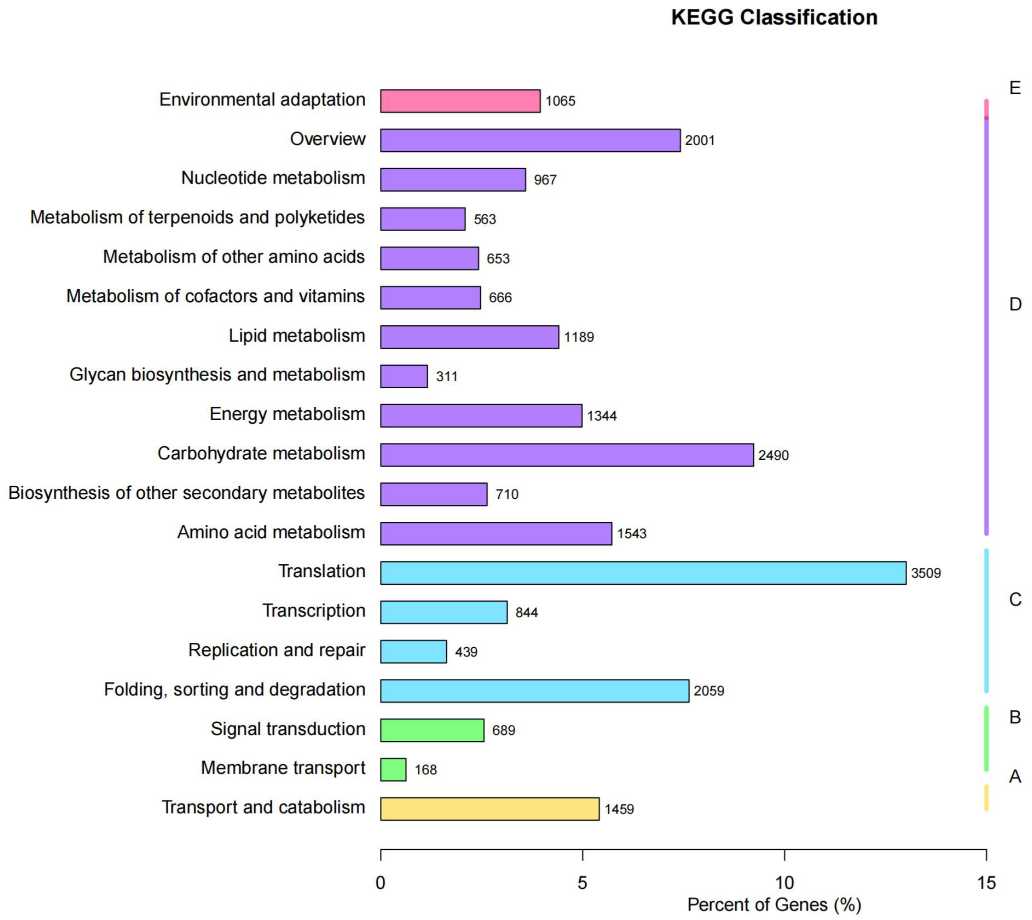


**Figure S3.** KEGG pathway classification of *C. oleifera* unigenes. A represents cellular processes, B represents environmental information processing, C represents geneticinformation processing, D represents metabolism, E represents organismal systems

**Table S1.** Distribution of unigene lengths in the transcriptome assembly

| Length Range (bp) | Number of Unigenes | Percentage (%) |
|---|---|---|
| 300~500 | 69, 175 | 40.77 |
| 501~1000 | 55, 785 | 32.88 |
| 1001~2000 | 31, 024 | 18.29 |
| >2000 | 13, 668 | 8.06 |

**Table S2.** Summary of functional annotation of *C. oleifera* unigenes

| Database | Number of Annotated Unigenes | Percentage (%) |
|---|---|---|
| NR | 19, 512 | 11.50 |
| NT | 53, 308 | 31.42 |
| KEGG (KO) | 26, 968 | 15.89 |
| Swiss-Prot | 52, 337 | 30.84 |
| Pfam | 100,023 | 58.95 |
| GO | 57, 706 | 34.01 |
| KOG | 19, 812 | 11.67 |
| Annotated in all databases | 2, 430 | 1.43 |
| Annotated in at least one database | 89, 569 | 52.79 |

**Table S3.** Functional classification of DEGs based on GO enrichment analysis

| GO accession | GO description | GO class | DEG Number | | | |
|---|---|---|---|---|---|---|
| | | | G1vs G0 | G2vs G0 | G3vs G0 | G4vs G0 |
| GO:0005618 | cell wall | Cellular component | 31 | 66 | 63 | 81 |
| GO:0030312 | external encapsulating structure | | 39 | 87 | 85 | 103 |
| GO:0048046 | apoplast | | 8 | 17 | 21 | 25 |
| GO:0009654 | photosystem II oxygen evolving complex | | 7 | 16 | 17 | 17 |
| GO:0071555 | cell wall organization | Biological process | 23 | 51 | 44 | 59 |
| GO:0045229 | external encapsulating structure organization | | 23 | 52 | 45 | 60 |
| GO:0071554 | cell wall organization or biogenesis | | 27 | 69 | 63 | 82 |
| GO:0042545 | cell wall modification | | 12 | 28 | 26 | 34 |
| GO:0006073 | cellular glucan metabolic process | | 30 | 87 | 98 | 118 |
| GO:0044042 | glucan metabolic process | | 30 | 87 | 98 | 118 |
| GO:0044264 | cellular polysaccharide metabolic process | | 30 | 90 | 101 | 124 |
| GO:0005976 | polysaccharide metabolic process | | 31 | 92 | 104 | 128 |
| GO:0044262 | cellular carbohydrate metabolic process | | 38 | 125 | 134 | 165 |

| | | | | | | |
|---|---|---|---|---|---|---|
| GO:0055114 | oxidation-reduction process | | 137 | 541 | 607 | 724 |
| GO:0005199 | structural constituent of cell wall | | 10 | 18 | 14 | 19 |
| GO:0020037 | heme binding | | 43 | 145 | 151 | 173 |
| GO:0046906 | tetrapyrrole binding | | 43 | 149 | 159 | 177 |
| GO:0016747 | transferase activity, transferring acyl groups other than amino-acyl groups | | 36 | 89 | 102 | 122 |
| GO:0030599 | pectinesterase activity | | 12 | 28 | 26 | 34 |
| GO:0016762 | xyloglucan:xyloglucosyl transferase activity | Molecular function | 8 | 17 | 21 | 25 |
| GO:0016684 | oxidoreductase activity, acting on peroxide as acceptor | | 17 | 62 | 49 | 69 |
| GO:0004601 | peroxidase activity | | 16 | 57 | 43 | 63 |
| GO:0016491 | oxidoreductase activity | | 133 | 554 | 623 | 741 |
| GO:0005506 | iron ion binding | | 30 | 95 | 120 | 127 |
| GO:0016209 | antioxidant activity | | 19 | 81 | 71 | 90 |

**Table S4.** KEGG pathway enrichment analysis of DEGs across germination stages

| Pathway | KEGG ID | DEG Number | | | |
|---|---|---|---|---|---|
| | | G1vs G0 | G2vs G0 | G3vs G0 | G4vs G0 |
| Phenylpropanoid biosynthesis | ko00940 | 40 | 102 | 93 | 122 |
| Photosynthesis - antenna proteins | ko00196 | 16 | 36 | 38 | 36 |
| Flavonoid biosynthesis | ko00941 | 19 | 35 | 38 | 40 |
| Phenylalanine metabolism | ko00360 | 20 | 29 | 31 | 37 |
| Photosynthesis | ko00195 | 16 | 37 | 41 | 38 |
| Starch and sucrose metabolism | ko00500 | 25 | 71 | 80 | 106 |
| Pentose and glucuronate interconversions | ko00040 | 13 | 42 | 37 | 52 |
| Plant hormone signal transduction | ko04075 | 20 | 50 | 83 | 88 |
| Stilbenoid, diarylheptanoid and gingerol biosynthesis | ko00945 | 6 | 23 | 24 | 27 |
| Cutin, suberine and wax biosynthesis | ko00073 | 3 | 15 | 14 | 18 |

**Table S5.** DEGs of phenylpropanoid biosynthesis across germination stages

| Gene ID | Gene Family | G0 | G1 | G2 | G3 | G4 | Product |
|---|---|---|---|---|---|---|---|
| Cluster-53230.12349 | PAL | 11.45 | 85.59 | 1157.45 | 1372.53 | 1240.22 | phenylalanine ammonia-lyase |
| Cluster-53230.93549 | PAL | 43.47 | 549.27 | 2369.92 | 3201.07 | 3490.49 | phenylalanine ammonia-lyase |
| Cluster-53230.93551 | PAL | 40.12 | 278.14 | 1419.73 | 1827.14 | 2039.26 | phenylalanine ammonia-lyase |
| Cluster-53230.93553 | PAL | 12.64 | 92.22 | 546.69 | 664.68 | 867.48 | phenylalanine ammonia-lyase |
| Cluster-53230.26136 | PAL | 24.33 | 455.44 | 1342.47 | 1794.99 | 2368.01 | phenylalanine ammonia-lyase |
| Cluster-53230.8268 | POD | 3.93 | 85.95 | 136.71 | 256.88 | 304.52 | peroxidase |
| Cluster-53230.8942 | POD | 1.12 | 50.87 | 364.01 | 375.92 | 567.49 | peroxidase |
| Cluster-53230.22917 | 4CL | 16.37 | 105.16 | 895.67 | 932.26 | 1248.34 | 4-coumarate--CoA ligase |
| Cluster-53230.98042 | 4CL | 54.48 | 1003.46 | 2044.84 | 3407.93 | 3773.03 | 4-coumarate--CoA ligase |
| Cluster-53230.12676 | bglB | 43.56 | 334.20 | 581.63 | 597.09 | 642.35 | beta-glucosidase |
| Cluster-53230.60336 | COMT | 204.18 | 1211.15 | 5475.22 | 7351.27 | 7813.31 | caffeoyl-CoA O-methyltransferase |

**Table S6.** DEGs of photosynthesis - antenna proteins and photosynthesis across germination stages

| Gene ID | Gene Family | G0 | G1 | G2 | G3 | G4 | Product |
|---|---|---|---|---|---|---|---|
| Cluster-53230.91274 | LHCA1 | 10.41 | 743.33 | 1624.60 | 2659.39 | 1411.49 | light-harvesting complex I chlorophyll a/b binding protein 1 |
| Cluster-53230.94801 | LHCA2 | 6.25 | 321.83 | 1233.93 | 2818.22 | 1379.97 | light-harvesting complex I chlorophyll a/b binding protein 2 |
| Cluster-53230.94802 | LHCA2 | 2.12 | 153.79 | 377.10 | 693.20 | 441.13 | light-harvesting complex I chlorophyll a/b binding protein 2 |
| Cluster-53230.11357 | LHCA3 | 0.00 | 33.04 | 79.97 | 189.91 | 75.98 | light-harvesting complex I chlorophyll a/b binding protein 3 |
| Cluster-53230.56169 | LHCA4 | 62.61 | 352.25 | 688.50 | 1712.62 | 797.74 | light-harvesting complex I chlorophyll a/b binding protein 4 |
| Cluster-53230.11564 | LHCB1 | 1.13 | 235.66 | 317.06 | 479.33 | 241.03 | light-harvesting complex II chlorophyll a/b binding protein 1 |
| Cluster-53230.94756 | LHCB1 | 7.69 | 2521.77 | 3712.57 | 6087.92 | 3344.28 | light-harvesting complex II chlorophyll a/b binding protein 1 |
| Cluster-53230.94757 | LHCB1 | 0.56 | 98.25 | 243.28 | 749.24 | 311.68 | light-harvesting complex II chlorophyll a/b binding protein 1 |
| Cluster-53230.94758 | LHCB1 | 9.28 | 2445.27 | 6459.04 | 12166.32 | 4605.14 | light-harvesting complex II chlorophyll a/b binding protein 1 |
| Cluster-53230.94759 | LHCB1 | 1.41 | 309.47 | 1575.83 | 3502.01 | 1238.72 | light-harvesting complex II chlorophyll a/b binding protein 1 |
| Cluster-53230.62003 | LHCB2 | 351.82 | 2710.18 | 7797.41 | 16781.75 | 7401.61 | light-harvesting complex II chlorophyll a/b binding protein 2 |
| Cluster-53230.69598 | LHCB2 | 1.69 | 38.15 | 124.88 | 307.63 | 104.52 | light-harvesting complex II chlorophyll a/b binding protein 2 |
| Cluster-53230.97181 | LHCB3 | 1.75 | 251.05 | 489.56 | 924.83 | 497.35 | light-harvesting complex II chlorophyll a/b binding protein 3 |
| Cluster-53230.18997 | LHCB4 | 72.68 | 385.30 | 577.81 | 1158.07 | 492.47 | light-harvesting complex II chlorophyll a/b binding protein 4 |
| Cluster-53230.98311 | LHCB5 | 5.92 | 263.38 | 799.39 | 1351.02 | 626.05 | light-harvesting complex II chlorophyll a/b binding protein 5 |
| Cluster-53230.95907 | LHCB6 | 39.80 | 811.94 | 1771.22 | 4525.33 | 2193.51 | light-harvesting complex II chlorophyll a/b binding protein 6 |
| Cluster-53230.62995 | psaD | 243.32 | 1516.83 | 3212.15 | 4814.01 | 3214.94 | photosystem I subunit II |
| Cluster-53230.23965 | psaE | 10.17 | 135.03 | 386.29 | 636.48 | 370.83 | photosystem I subunit IV |
| Cluster-53230.75849 | psaF | 146.46 | 1359.53 | 3094.83 | 5766.87 | 3220.16 | photosystem I subunit III |
| Cluster-53230.98424 | psaH | 12.16 | 132.70 | 395.03 | 836.67 | 601.42 | photosystem I subunit VI |
| Cluster-53230.10426 | psaO | 8.12 | 234.00 | 1301.99 | 2678.48 | 1099.62 | photosystem I subunit PsaO |
| Cluster-53230.89975 | psaK | 5.73 | 135.66 | 296.69 | 548.89 | 221.44 | photosystem I subunit X |
| Cluster-53230.98745 | psaN | 25.12 | 170.01 | 420.91 | 924.05 | 348.49 | photosystem I subunit PsaN |
| Cluster-53230.45580 | psbO | 256.00 | 1208.80 | 3950.15 | 7566.46 | 4060.08 | photosystem II oxygen-evolving enhancer protein 1 |

| | | | | | | | |
|---|---|---|---|---|---|---|---|
| Cluster-53230.90899 | psbO | 7.49 | 149.78 | 616.76 | 1967.20 | 877.08 | photosystem II oxygen-evolving enhancer protein 1 |
| Cluster-53230.90900 | psbO | 5.52 | 95.17 | 268.84 | 767.15 | 300.91 | photosystem II oxygen-evolving enhancer protein 1 |
| Cluster-53230.100978 | psbW | 16.10 | 111.52 | 531.35 | 1261.72 | 641.04 | photosystem II PsbW protein |
| Cluster-53230.12869 | psbY | 5.34 | 112.55 | 870.49 | 2366.15 | 1003.73 | photosystem II PsbY protein |
| Cluster-53230.55967 | psbY | 40.75 | 270.94 | 665.27 | 1393.66 | 570.32 | photosystem II PsbY protein |
| Cluster-53230.8278 | psb27 | 3.96 | 102.73 | 134.68 | 422.50 | 223.04 | photosystem II Psb27 protein |
| Cluster-53230.3694 | psb28 | 1.12 | 59.45 | 162.74 | 415.73 | 210.21 | photosystem II 13kDa protein |

**Table S7.** DEGs of flavonoid biosynthesis across germination stages

| Gene ID | Gene Family | G0 | G1 | G2 | G3 | G4 | Product |
|---|---|---|---|---|---|---|---|
| Cluster-53230.12842 | CHS | 8.74 | 2547.68 | 5324.91 | 11362.35 | 11519.41 | chalcone synthase |
| Cluster-53230.12843 | CHS | 0.56 | 324.82 | 840.94 | 1963.02 | 2330.95 | chalcone synthase |
| Cluster-53230.11702 | CHS | 5.80 | 852.76 | 2628.72 | 5664.77 | 5701.02 | chalcone synthase |
| Cluster-53230.98814 | ANR | 11.67 | 381.22 | 1015.79 | 2405.94 | 2526.70 | anthocyanidin reductase |
| Cluster-53230.98815 | ANR | 4.82 | 509.25 | 1329.28 | 3235.21 | 3604.78 | anthocyanidin reductase |
| Cluster-53230.15757 | F3H | 130.11 | 811.92 | 1911.81 | 3869.79 | 4260.88 | naringenin 3-dioxygenase |
| Cluster-53230.98550 | ANS | 8.46 | 1275.04 | 3466.38 | 8755.31 | 9025.55 | leucoanthocyanidin dioxygenase |
| Cluster-53230.12442 | DFR | 8.76 | 611.01 | 1549.35 | 1966.93 | 1935.85 | bifunctional dihydroflavonol 4-reductase/flavanone 4-reductase |

**Table S8.** DEGs of starch and sucrose metabolism across germination stages

| Gene ID | Gene Family | G0 | G1 | G2 | G3 | G4 | Product |
|---|---|---|---|---|---|---|---|
| Cluster-53230.108221 | sacA | 0.00 | 25.59 | 37.41 | 90.07 | 90.85 | beta-fructofuranosidase |
| Cluster-53230.29630 | GAE | 5.95 | 178.73 | 382.05 | 424.66 | 696.71 | UDP-glucuronate 4-epimerase |
| Cluster-53230.45432 | SUS | 455.85 | 8214.25 | 20131.56 | 26756.09 | 30828.47 | sucrose synthase |
| Cluster-53230.66369 | scrK | 467.90 | 3322.85 | 2642.71 | 2372.97 | 1671.90 | fructokinase |

**Table S9.** DEGs of plant hormone signal transduction across germination stages

| Gene ID | Gene Family | G0 | G1 | G2 | G3 | G4 | Product |
|---|---|---|---|---|---|---|---|
| Cluster-53230.4822 | IAA | 24.49 | 205.63 | 375.69 | 444.79 | 499.67 | auxin-responsive protein IAA |
| Cluster-53230.95594 | IAA | 43.63 | 497.30 | 819.16 | 1181.80 | 1238.59 | auxin-responsive protein IAA |
| Cluster-53230.30072 | AUX1, LAX | 116.06 | 667.61 | 1452.59 | 3091.57 | 3299.22 | auxin influx carrier (AUX1 LAX family) |
| Cluster-53230.10248 | AUX1, LAX | 34.16 | 181.84 | 202.40 | 442.87 | 490.37 | auxin influx carrier (AUX1 LAX family) |
| Cluster-53230.10041 | ABF | 15.54 | 96.58 | 107.85 | 123.89 | 137.97 | ABA responsive element binding factor |
| Cluster-53230.106471 | MYC2 | 6.54 | 59.51 | 161.17 | 309.18 | 360.92 | transcription factor MYC2 |
| Cluster-53230.46125 | CYCD3 | 145.84 | 785.07 | 1196.41 | 1568.81 | 1778.87 | cyclin D3, plant |

**Table S10.** DEGs of TF across germination stages

| Gene ID | Gene Family | G0 | G1 | G2 | G3 | G4 | Product |
|---|---|---|---|---|---|---|---|
| Cluster-53230.13403 | AP2/ERF-ERF | 20.99 | 267.81 | 506.90 | 727.18 | 803.12 | PREDICTED: Solanum pennellii ethylene-responsive transcription factor 12 (LOC107010420), mRNA |
| Cluster-53230.39139 | AP2/ERF-ERF | 2885.26 | 639.04 | 497.17 | 205.23 | 109.70 | Camellia sinensis cultivar Yingshuang ethylene responsive element binding factor (ERF-B4) mRNA, complete cds |
| Cluster-53230.14735 | AUX/IAA | 62.22 | 306.65 | 1226.39 | 1352.39 | 1920.17 | PREDICTED: Jatropha curcas auxin-responsive protein IAA26-like (LOC105631536), mRNA |
| Cluster-51114.0 | B3 | 5.93 | 68.22 | 88.81 | 103.05 | 150.47 | PREDICTED: Jatropha curcas putative B3 domain-containing protein At5g58280 (LOC105638609), transcript variant X2, mRNA |
| Cluster-53230.109578 | B3 | 0.58 | 40.81 | 58.86 | 71.33 | 79.44 | PREDICTED: Vigna angularis B3 domain-containing protein At5g42700-like (LOC108343369), mRNA |
| Cluster-53230.671 | B3 | 2.52 | 59.04 | 98.20 | 101.41 | 157.94 | PREDICTED: Sesamum indicum B3 domain-containing protein At5g42700-like (LOC105166259), transcript variant X2, mRNA |
| Cluster-53230.99007 | bHLH | 14.13 | 132.27 | 439.10 | 509.36 | 531.82 | Camellia sinensis bHLH transcription factor (GL3) mRNA, complete cds |
| Cluster-53230.63588 | C2C2-GATA | 140.10 | 775.28 | 1108.23 | 1850.82 | 2399.48 | Camellia sinensis DNA, SSR marker, MSE0231 |
| Cluster-53230.17237 | C2H2 | 2.82 | 51.00 | 93.56 | 145.98 | 161.15 | PREDICTED: Citrus sinensis zinc finger protein ZAT5-like (LOC102630422), mRNA |
| Cluster-53230.8774 | CPP | 18.03 | 229.50 | 252.23 | 258.34 | 343.79 | PREDICTED: Vitis vinifera protein tesmin/TSO1-like CXC 2 (LOC100265943), transcript variant X1, mRNA |
| Cluster-53230.25530 | FAR1 | 6.06 | 82.83 | 97.44 | 122.81 | 206.81 | Protein FAR1-RELATED SEQUENCE 7 OS=Arabidopsis thaliana GN=FRS7 PE=2 SV=1 |
| Cluster-38499.0 | GRAS | 3.08 | 62.96 | 85.45 | 114.57 | 136.60 | PREDICTED: Vitis vinifera scarecrow-like protein 28 (LOC100247949), mRNA |
| Cluster-53230.76820 | GRAS | 5.67 | 174.99 | 210.80 | 272.20 | 279.26 | PREDICTED: Ziziphus jujuba scarecrow-like protein 28 (LOC107430006), mRNA |
| Cluster-53230.98768 | HB-WOX | 0.28 | 34.39 | 201.08 | 426.50 | 648.60 | PREDICTED: Glycine max WUSCHEL-related homeobox 4 (LOC100500674), mRNA |

<table>
<tr><td>Cluster-53230.46793</td><td>HMG</td><td>89.46</td><td>632.88</td><td>669.14</td><td>1093.39</td><td>1296.93</td><td>PREDICTED: Gossypium arboreum high mobility group B protein 7 (LOC108477080), mRNA</td></tr>
<tr><td>Cluster-53230.25867</td><td>LIM</td><td>125.85</td><td>760.75</td><td>1353.45</td><td>1347.43</td><td>1420.42</td><td>PREDICTED: Juglans regia LIM domain-containing protein WLIM2b-like (LOC109002721), transcript variant X9, mRNA</td></tr>
<tr><td>Cluster-53230.95526</td><td>MYB</td><td>8.74</td><td>73.56</td><td>137.00</td><td>188.78</td><td>215.08</td><td>PREDICTED: Pyrus x bretschneideri transcription factor MYB32-like (LOC103932535), transcript variant X2, mRNA</td></tr>
<tr><td>Cluster-53230.10552</td><td>MYB-related</td><td>16.10</td><td>120.98</td><td>296.05</td><td>378.45</td><td>465.90</td><td>PREDICTED: Theobroma cacao transcription factor MYB86 (LOC18603988), mRNA</td></tr>
<tr><td>Cluster-53230.2966</td><td>NAC</td><td>1.13</td><td>37.68</td><td>58.60</td><td>92.88</td><td>102.01</td><td>PREDICTED: Ricinus communis NAC domain-containing protein 90 (LOC8271946), mRNA</td></tr>
<tr><td>Cluster-53230.89</td><td>NF-YC</td><td>2.55</td><td>161.35</td><td>128.68</td><td>152.33</td><td>188.37</td><td>Eutrema salsugineum hypothetical protein (EUTSA_v10003290mg) mRNA, complete cds</td></tr>
<tr><td>Cluster-53230.26712</td><td>Others</td><td>46.08</td><td>321.34</td><td>491.10</td><td>795.93</td><td>812.07</td><td>Glycine max uncharacterized LOC100305720 (LOC100305720), mRNA >gb|BT089473.1| Soybean clone JCVI-FLGm-2E17 unknown mRNA</td></tr>
<tr><td>Cluster-53230.9336</td><td>Others</td><td>15.84</td><td>242.21</td><td>815.85</td><td>939.03</td><td>1049.28</td><td>PREDICTED: Eucalyptus grandis zinc finger protein CONSTANS-LIKE 5 (LOC104435984), mRNA</td></tr>
<tr><td>Cluster-47443.0</td><td>SBP</td><td>1.96</td><td>35.95</td><td>64.56</td><td>97.48</td><td>102.71</td><td>Morus notabilis Squamosa promoter-binding-like protein 8 partial mRNA</td></tr>
<tr><td>Cluster-53230.67030</td><td>SBP</td><td>48.02</td><td>291.98</td><td>243.71</td><td>196.77</td><td>161.85</td><td>PREDICTED: Nelumbo nucifera squamosa promoter-binding protein 1 (LOC104599433), mRNA</td></tr>
<tr><td>Cluster-53230.36647</td><td>SET</td><td>63.63</td><td>255.33</td><td>336.22</td><td>319.70</td><td>426.93</td><td>PREDICTED: Ziziphus jujuba histone-lysine N-methyltransferase CLF (LOC107424507), transcript variant X1, mRNA</td></tr>
<tr><td>Cluster-53230.91358</td><td>SNF2</td><td>44.38</td><td>388.78</td><td>372.72</td><td>536.87</td><td>725.29</td><td>PREDICTED: Vitis vinifera ATP-dependent DNA helicase DDM1 (LOC100263333), transcript variant X2, mRNA</td></tr>
<tr><td>Cluster-53230.102097</td><td>SRS</td><td>12.39</td><td>91.48</td><td>183.62</td><td>329.71</td><td>454.88</td><td>PREDICTED: Ricinus communis protein LATERAL ROOT PRIMORDIUM 1 (LOC8268536), mRNA</td></tr>
</table>

**Table S11.** Specific primers of selected genes for qRT-PCR analysis

| Gene ID | Sequence |
|---|---|
| Cluster-53230.26136-S | AGGTAGCCATTTGGATGAGGTG |
| Cluster-53230.26136-A | TTTGAAGAGCAGCACCTTGATT |
| Cluster-53230.98042-S | TCCCGACATACCCATCTCCA |
| Cluster-53230.98042-A | CCCATGAATGCGAAGACGAA |
| Cluster-53230.62003-S | TGGGCTTCATTGAGGGCTAC |
| Cluster-53230.62003-A | AGCGAGATGGTCCAAGAGGTTC |
| Cluster-53230.45580-S | AGAAAGACGAACCACGGTATGAT |
| Cluster-53230.45580-A | GGAGAAAGATGGAATTGACTACGC |
| Cluster-53230.99007-S | AATGTTGTTGGCGTAGTTATCGG |
| Cluster-53230.99007-A | GAGAAGGGTTGAGGAGTTGGATT |
| Cluster-53230.10248-S | CCCAAGGCTATGAAGACAAGGA |
| Cluster-53230.10248-A | ATGAGTATGGCAATGTCAGCAAC |
| Cluster-53230.46125-S | GTTCTGGGAAGACGAAGAGTTGA |
| Cluster-53230.46125-A | GAGAATGAGTAGTGGGCGTTGA |
| Cluster-53230.4822-S | CAGGCAACAGTTTCTCAAGGATA |
| Cluster-53230.4822-A | CTTCGGAACAACCACAACCATT |
| Cluster-53230.11702-S | CGCTTGATGCTAGGCAGGAC |
| Cluster-53230.11702-A | GCCACCAGCAAAGCAACCT |
| Tubulin-F | CCATGCCTTGGATCACATTT |

| | |
|---|---|
| Tubulin-R | TGGGGCCATTAATGTAGACG |

**Table S12.** Verification of selected differentially expressed genes using qRT-PCR

| Selected genes | Relative expression | | | | |
|---|---|---|---|---|---|
| | G0 | G1 | G2 | G3 | G4 |
| Cluster-53230.26136（PAL） | 0.73 | 0.85 | 0.80 | 1.09 | 1.23 |
| Cluster-53230.98042（4CL） | 0.52 | 1.02 | 0.95 | 0.74 | 0.97 |
| Cluster-53230.62003（LHCB2） | 0.92 | 1.08 | 0.94 | 1.22 | 1.25 |
| Cluster-53230.45580（psbO） | 0.69 | 0.89 | 0.92 | 1.16 | 0.99 |
| Cluster-53230.99007（bHLH） | 0.54 | 0.88 | 0.76 | 0.79 | 0.65 |
| Cluster-53230.10248（AUX1, LAX） | 0.55 | 1.04 | 0.70 | 0.98 | 0.63 |
| Cluster-53230.46125（CYCD3） | 0.62 | 0.96 | 0.67 | 0.90 | 0.82 |
| Cluster-53230.4822（IAA） | 0.91 | 1.81 | 0.81 | 1.15 | 1.18 |
| Cluster-53230.11702（CHS） | 0.78 | 1.06 | 0.86 | 1.03 | 0.93 |
